# The Role of Bone-Derived Hormones in Glucose Metabolism, Diabetic Kidney Disease, and Cardiovascular Disorders

**DOI:** 10.3390/ijms23042376

**Published:** 2022-02-21

**Authors:** Yuichi Takashi, Daiji Kawanami

**Affiliations:** Department of Endocrinology and Diabetes Mellitus, Fukuoka University School of Medicine, 7-45-1 Nanakuma, Jonan-ku, Fukuoka 814-0180, Japan; kawanami@fukuoka-u.ac.jp

**Keywords:** diabetes, diabetic kidney disease, cardiovascular disorders, bone-derived hormone, fibroblast growth factor 23, osteocalcin, sclerostin, lipocalin 2

## Abstract

Bone contributes to supporting the body, protecting the central nervous system and other organs, hematopoiesis, the regulation of mineral metabolism (mainly calcium and phosphate), and assists in respiration. Bone has many functions in the body. Recently, it was revealed that bone also works as an endocrine organ and secretes several systemic humoral factors, including fibroblast growth factor 23 (FGF23), osteocalcin (OC), sclerostin, and lipocalin 2. Bone can communicate with other organs via these hormones. In particular, it has been reported that these bone-derived hormones are involved in glucose metabolism and diabetic complications. Some functions of these bone-derived hormones can become useful biomarkers that predict the incidence of diabetes and the progression of diabetic complications. Furthermore, other functions are considered to be targets for the prevention or treatment of diabetes and its complications. As is well known, diabetes is now a worldwide health problem, and many efforts have been made to treat diabetes. Thus, further investigations of the endocrine system through bone-derived hormones may provide us with new perspectives on the prediction, prevention, and treatment of diabetes. In this review, we summarize the role of bone-derived hormones in glucose metabolism, diabetic kidney disease, and cardiovascular disorders.

## 1. Introduction

Diabetes has become a major social health problem throughout the world. Diabetes is caused by both insufficiency of insulin secretion from pancreatic β cells (mainly type 1 diabetes) and increased insulin resistance in the liver and peripheral tissues due to the presence of lifestyle factors including obesity, lack of exercise, and an unbalanced diet (mainly type 2 diabetes). In addition, diabetic complications have been characterized by micro- or macrovascular dysfunction. However, the concept of diabetic complications has become extensive and complicated [1]. For instance, bone disorders have been drawing attention as a novel diabetic complication, and accumulating evidence supports that diabetes is associated with an increased risk of osteoporotic fracture [2,3,4,5,6,7,8,9]. To date, numerous efforts have been made to predict, prevent, and treat both diabetes and diabetic complications.

It has been revealed that bone secretes several hormones and systemically regulates the functions of other organs [10]. Currently, fibroblast growth factor 23 (FGF23) [11,12,13], osteocalcin (OC) [14], sclerostin [15], and lipocalin 2 [16] have been identified as bone-derived humoral factors (Table 1). Importantly, these bone-derived hormones are involved in glucose metabolism and diabetic complications. Diabetes is considered to be a chronic disease with pathophysiological manifestations in pancreatic β cells and α cells, liver, muscle, adipose tissue, intestine, the central and peripheral nervous system, kidneys, and the vascular system [17]. In addition, bone has been suggested to be another target organ that modulates glucose metabolism and diabetic complications. Therefore, these bone-derived hormones can become useful biomarkers for predicting the incidence of diabetes and the progression of diabetic complications. Furthermore, the effects of bone-derived hormones are considered to be targets for the prevention or treatment of diabetes and diabetic complications. Lately, several results of prospective clinical studies have been reported, and the clinical significance of bone-derived hormones in humans is gradually accumulating. Thus, a concise review covering the broad range of findings between bone-derived hormones and diabetes is currently needed. This review focused on new insights into every bone-derived hormone—FGF23, OC, sclerostin, and lipocalin 2—from the findings of both experimental and clinical studies. In this review, we aimed to summarize the role of bone-derived hormones in glucose metabolism., diabetic kidney disease, and cardiovascular disorders.

## 2. Fibroblast Growth Factor 23 (FGF23)

### 2.1. The Regulation of Glucose Metabolism by FGF23

FGF23 was the first bone-derived hormone to be reported. FGF23 was identified as a gene or a humoral factor responsible for hypophosphatemic rickets/osteomalacia [12,13]. FGF23 is produced by bone, especially by osteoblasts and osteocytes [18,19], and it mainly works at the renal proximal tubules via the FGF receptor (FGFR) 1c/α-Klotho complex [20,21] and regulates phosphate and vitamin D metabolism [22]. FGF23 inhibits the reabsorption of phosphate in the renal proximal tubules by suppressing the expression of type Ⅱa and Ⅱc sodium–phosphate cotransporters (NaPis) encoded by *SLC34A1* and *SLC34A3*, respectively [22]. At the same time, FGF23 reduces the blood 1,25-dihydroxyvitamin D [1,25(OH)_2_D] level by suppressing the expression of *CYP27B1* and enhancing the expression of *CYP24A1* [22]. *CYP27B1* and *CYP24A1* encode 25-hydroxyvitamin D-1α-hydroxylase and 25-hydroxyvitamin D-24-hydroxylase, respectively. Because 1,25(OH)_2_D enhances the expression of NaPi-Ⅱb, which is encoded by *SLC34A2* in the intestine, FGF23 reduces intestinal phosphate absorption by suppressing the 1,25(OH)_2_D level and the expression of NaPi-Ⅱb [22]. Finally, FGF23 works to reduce the blood phosphate level. FGF23 can bind to FGFR1c in the presence of α-Klotho [20,21]. α-Klotho is expressed in several restricted tissues including the kidney and parathyroid glands [23]. Recently, the crystal structure of the FGFR1c/α-Klotho complex was revealed, and α-Klotho was reported to be a nonenzymatic molecular scaffold for FGF23 signaling [24]. Since FGF23 is a phosphotropic hormone, it could be considered that phosphate modulates the production of FGF23 and the blood level of FGF23. A high phosphate diet increases the blood’s FGF23 level, and high extracellular phosphate enhances the production of FGF23 [25,26,27]. Recently, we revealed that the activation of unliganded FGFR1c by extracellular phosphate regulates the production of FGF23 [28]. In addition, other factors, such as inflammatory cytokines [29,30], iron deficiency [31,32], hypoxia [33], erythropoietin [34], and aldosterone [35] were reported to affect the blood’s FGF23 level; however, the precise mechanism and significance of these factors are unclear [36].

Hypophosphatemia was reported to be the cause of poor glycemic control by decreased insulin secretion due to the fact of impaired adenosine triphosphate (ATP) production in pancreatic islets [37,38]. Furthermore, phosphate supplementation improved insulin sensitivity in both glucose-intolerant hypophosphatemic patients and healthy individuals [39,40]. Other studies suggested that blood levels of phosphate were lower in patients with diabetes and a preserved kidney function in comparison to healthy controls without diabetes [41]. Moreover, increased blood phosphate levels are associated with improved glycemic control in patients with diabetes [42,43]. Therefore, the blood phosphate level is disturbed in the early progression of diabetes and phosphate deregulation adversely affects glucose metabolism [44]. FGF23, which contributes to the regulation of the blood phosphate level, also influences glucose metabolism. In mice, ablation of the *Fgf23* gene results in hypoglycemia and increased peripheral insulin sensitivity [45,46]. However, the effects of FGF23 on glucose metabolism in humans are less known. Several studies indicated a negative association between blood FGF23 levels and insulin sensitivity [47,48,49,50]. Moreover, FGF23 is positively associated with resistin, which is a regulator of insulin resistance [51]. Whether FGF23 induces insulin resistance should be further addressed.

The impact of FGF23 on diabetes is quite complex. In some studies, diabetes was associated with higher blood FGF23 levels [49,50,52], whereas other studies did not find these associations [53,54]. Recently, it was reported that insulin or insulin-like growth factor 1 (IGF-1)-dependent phosphoinositide 3-kinase (PI3K)/protein kinase B (Akt)/forkhead box protein O1 (FOXO1) signaling suppresses the production of FGF23 [55]. In mice, insulin deficiency caused a surge in the blood’s FGF23 level. In addition, the administration of insulin reversed the elevation of the blood’s FGF23 level [55]. Furthermore, in women, an inverse association was observed between the area under the curve (AUC) of the insulin time curve reflecting insulin secretion during the first 60 min after an oral glucose tolerance test and the AUC of the FGF23 time curve during the same time period, reflecting the secreted FGF23 within 60 min, suggesting that insulin is a negative regulator of FGF23 [55]. Accordingly, blood FGF23 levels in type 1 diabetes patients with pathophysiological insulin deficiency may be high, and those in type 2 diabetes patients with hyperinsulinemia due to the fact of insulin resistance may be low. On the other hand, many studies demonstrated that type 2 diabetes was associated with higher blood FGF23 levels [49,50,52]. Moreover, inflammatory markers, such as C-reactive protein (CRP) and interleukin-6 (IL-6), are associated with FGF23 elevation [29,56,57]. Inflammation is a major trigger of FGF23 production [30] and most type 2 diabetes patients, especially those with obesity, suffer from inflammatory conditions [58]. A chronic inflammatory state may overrule the suppressive effect of hyperinsulinemia in patients with type 2 diabetes, resulting in their higher blood FGF23 levels. Furthermore, an oral glucose load in vitamin-D-deficient patients with impaired glucose metabolism decreased the blood’s FGF23 level, which cannot be attributed to a change in the insulin level [59]. On the other hand, a diet-induced increase in the blood’s FGF23 level did not affect fasting glucose or insulin levels [59]. The underlying mechanism by which a glucose load changes the blood’s FGF23 level is unknown. Furthermore, patients with diabetes are more prone to develop early tubular injury, prior to a measurable decrease in kidney function or albuminuria [60]. This early tubular dysfunction could, at least, partly contribute to the higher blood FGF23 levels in patients with diabetes [53]. Thus, we considered potential treatment methods to modulate abnormalities in phosphate and FGF23.

### 2.2. FGF23 in Diabetic Complications

Recent reports showed that a higher blood FGF23 level is a novel predictor of all-cause and cardiovascular mortality in patients with type 2 diabetes [61,62,63,64,65,66]. The associations restricted to patients with an estimated glomerular filtration rate (eGFR) of >60 mL/min/1.73 m^2^ persisted after adjustment for eGFR, linking a higher FGF23 level with adverse outcomes, even in patients without impaired kidney function [65]. Although the risk may be relevant in diabetic patients with a preserved kidney function, it is strongly accentuated in patients with diabetic nephropathy. Another study reported that FGF23 is a significant independent predictor of the renal outcome in patients with macroalbuminuric diabetic nephropathy [61]. Furthermore, in db/db mice, which are an animal model of type 2 diabetes, blocking of FGF23 action by the administration of the C-terminal tail of FGF23 improved diabetic nephropathy by decreasing inflammation and fibrosis without changing the blood levels of phosphate or FGF23 [67,68]. It was revealed that blood FGF23 levels started to increase early in the progression of chronic kidney disease (CKD), before the increase in the blood phosphate level [69]. Patients with type 2 diabetes may be susceptible to abnormalities in bone and mineral metabolism including increased blood FGF23 levels [70]. Furthermore, high dietary phosphate intake is also associated with high blood FGF23 levels [25,26,28,71]. As mentioned above, FGF23 may promote insulin resistance, which may influence the risk of adverse outcomes. Especially under CKD conditions, FGF23 was reported to affect various adverse events including left ventricular hypertrophy (LVH) [72], vascular calcification [73,74], impairment of neutrophil activation [75], induction of the expression of tumor necrosis factor α (TNFα) in the macrophages [76], and stimulation of the secretion of inflammatory cytokines in the liver [77]. While these off-target actions of FGF23 also affect adverse outcomes, the precise mechanisms underlying these effects are still unclear. For instance, FGF23 was shown to cause pathological cardiac hypertrophy through FGFR4 [78]. Because cardiomyocytes do not express α-Klotho and FGF23 showed quite a low affinity for FGFRs [20,23,79], the mechanism by which FGF23 activates FGFR4 in cardiomyocytes without α-Klotho remains to be clarified. Future studies should clarify whether FGF23 is merely a disease severity marker or a contributor to adverse outcomes in type 2 diabetes and establish whether antidiabetic medication can modify blood FGF23 levels.

## 3. Osteocalcin (OC)

### 3.1. The Regulation of Glucose Metabolism by OC

Osteocalcin (OC) is a bone-derived hormone directly associated with glucose metabolism [14]. OC is produced by osteoblasts, and mostly OC works as a non-collagen bone matrix protein with a high affinity for hydroxyapatite after carboxylation at three glutamate residues (17, 21, and 24 in humans) by γ-carboxylase [80]. Part of OC enters into systemic circulation. Because the blood level of OC reflects the bone formation ability of osteoblasts, OC is now used as a bone metabolism marker in clinical settings [81]. On the other hand, undercarboxylated-type OC was called undercarboxylated osteocalcin (ucOC) [80]. Unlike OC, ucOC does not stay in the bone and enters into systemic circulation. Although carboxylation of OC is dependent on vitamin K, the blood ucOC level increases in vitamin K deficiency [80]. Thus, we use ucOC as a marker to determine whether vitamin K preparations should be administered to patients with osteoporosis [82].

**Figure 1:**
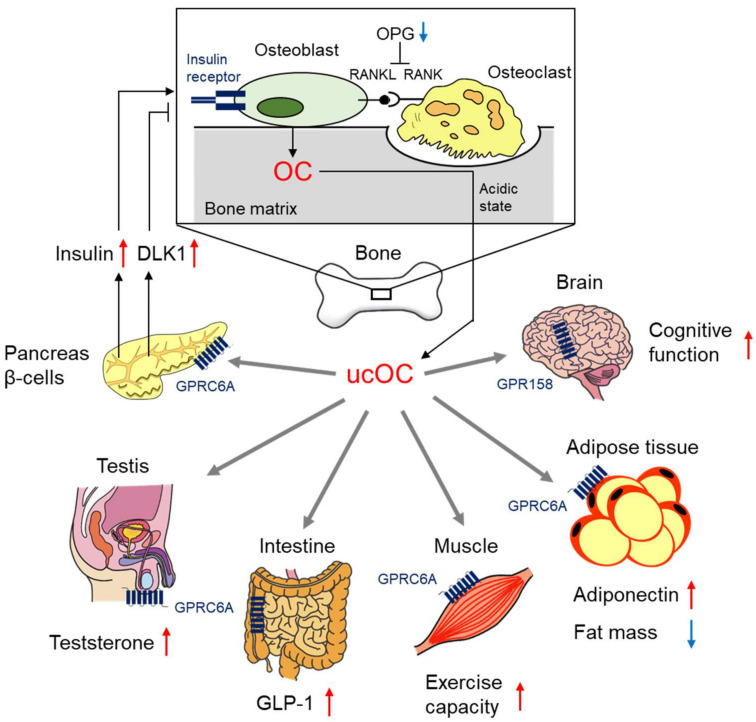
Osteocalcin (OC) affects glucose metabolism with various endocrine functions. OC is produced by osteoblasts via insulin receptor signaling. Stimulation of bone absorption by osteoclasts converts OC into undercarboxylated OC (ucOC), which is considered to be the endocrinologically active form of OC. UcOC affects pancreatic β cells, adipose tissue, testis, intestine, skeletal muscle, and brain. These various effects of OC are associated with glucose metabolism and diabetic complications.

Subsequently, it was reported that OC increases the secretion of insulin from pancreatic β cells and insulin sensitivity in peripheral tissues through experimental models using OC knockout mice [14]. A new concept of bone–glucose metabolism linkage was proposed in this report. Further experimental investigations revealed that insulin receptor signaling enhances the production of OC in osteoblasts [83,84]. The insulin receptor signaling in osteoblasts also suppresses osteoprotegerin (OPG), which leads to inhibition of the receptor activator of the NF-κB ligand (RANKL) action at the receptor activator of NF-κB (RANK) and enhances bone resorption via osteoclasts [83,84]. OC is converted into ucOC by acidic stimulation during bone resorption and is secreted into systemic circulation (Figure 1) [83,84]. ucOC is considered to be the endocrinologically active form of OC in experimental models [85]. In addition, GPRC6A was identified as the specific receptor of ucOC [86]. Circulatory ucOC stimulated the secretion of insulin from pancreatic β cells via this receptor [86]. Therefore, there is a positive feedback loop between bone and pancreatic islets. In addition, ucOC also enhances the production of delta like-1 (DLK1) in pancreatic β cells, and DLK1 inhibits the insulin signaling-dependent OC production in osteoblasts (Figure 1) [87].

It was revealed that ucOC has multiple functions (Figure 1), including enhancing the secretion of adiponectin from adipose tissue [88], reducing fat mass [88], enhancing the secretion of testosterone from the testis [89], enhancing the secretion of glucagon-like peptide-1 (GLP-1) from the intestine [90], enhancing the cognitive function in the brain [91], and enhancing the exercise capacity of skeletal muscle [92]. The receptor of ucOC is GPRC6A, which is broadly expressed in various organs [86,89,90,92,93] but not expressed in the brain [94,95]. G protein-coupled receptor 158 (GPR158) has been identified as the receptor of ucOC in the brain that is associated with cognitive function [96]. It could be considered that these various functions of ucOC may improve the pathophysiology of diabetes and prevent several complications of diabetes.

Since the discovery of the metabolic functions of OC in rodents [14], evidence to support the clinical significance of OC in humans is gradually accumulating. Many clinical studies have supported the results obtained using experimental models. Because most human studies were cross-sectional examinations, it remains inconclusive whether these beneficial effects of ucOC can also be found in humans. We also summarize human data concerning ucOC. First, we previously analyzed the association between the blood ucOC level and insulin secretion ability in patients with type 2 diabetes [97]. To evaluate the insulin secretion ability in detail, both a glucagon loading test and meal tolerance test were performed. As a result, their blood ucOC levels were positively associated with blood C-peptide levels stimulated by glucagon loading or meal intake [97]. These associations were strongly recognized in patients whose glycated hemoglobin (HbA1c) was <8.0%. Furthermore, the ratio of the patients who achieved an HbA1c value < 7.0% after 6 months in the higher ucOC group was significantly higher than that in the lower ucOC group (divided according to the median blood ucOC level in the subjects) [97]. Thus, it could be considered that ucOC may reflect the reserve capacity of the β-cell function and that ucOC may be associated with future glycemic control in patients with type 2 diabetes. In addition, it was indicated that ucOC enhanced the β-cell function in human islets from cadaveric donors [98]. According to large epidemiological studies, a higher blood ucOC level was associated with a reduced risk of diabetes in a community dwelling population [99,100]. In a longitudinal study, an increase in ucOC was associated with a decline in HOMA-IR over two years in a community dwelling population [101]. Recent prospective cohort studies demonstrated that lower OC levels are associated with an increased risk of incident diabetes [102,103,104,105]. Regarding the other functions of ucOC in humans, it was reported that the blood ucOC level was inversely associated with fat mass and positively associated with the blood adiponectin level [106], blood testosterone level [107], and cognitive function [108] in patients with type 2 diabetes.

On the other hand, several issues remain to be clarified. First, large-scale prospective clinical research concerning ucOC is currently insufficient. Second, it is unclear whether ucOC is the active form in humans. Clinical and epidemiological studies have assessed the associations of total OC with diabetes. The standard assay of OC detects both the carboxylated and uncarboxylated forms, indicating total OC. A meta-analysis showed that a higher total OC level was associated with an increase in HOMA-B and a decline in the fasting plasma glucose level, HbA1c, HOMA-IR, and body mass index (BMI) [109]. In addition, this analysis supported an inverse association of blood OC with the risk of adverse metabolic outcomes [109]. Third, there are few equivalent data on patients with type 1 diabetes. According to a previous report, the blood ucOC level in patients with type 1 diabetes is considered to be determined by the dose of exogenous insulin injection [110]. On the other hand, no studies have reported that various beneficial effects of ucOC, such as a reduction in fat mass, were also found in patients with type 1 diabetes [111,112]. We demonstrated an inverse association between blood ucOC or OC levels and body fat in patients with type 1 diabetes [113]. These associations remained significant even after adjustment for each factor such as sex, HbA1c, body weight-adjusted total daily dose of insulin, and the duration of diabetes. Thus, we considered that one of the beneficial effects of ucOC and OC was also observed in patients with type 1 diabetes. Further investigation in patients with type 1 diabetes is needed in the future.

### 3.2. OC in Diabetic Complications

Lately, a prospective cohort study with large samples has reported the association of baseline OC levels and risk of incident diabetic kidney disease [105]. Blood levels of ucOC and OC were significantly lower in patients with coronary artery disease and type 2 diabetes in comparison to patients with coronary artery disease without type 2 diabetes [114]. In addition, lower ucOC levels were associated with adverse cardiovascular risk in patients with coronary artery disease [115]. Thus, it is possible that OC can become a predictor of cardiovascular outcomes in patients with coronary artery disease. Another study using apolipoprotein E (ApoE) knockout mice, which is an animal model of atherosclerosis, further indicated that the administration of OC improved vascular endothelium-dependent relaxation by the increase in endothelial nitric oxide synthase (eNOS) phosphorylation and the activation of the PI3K/Akt signaling pathway [114]. Thus, OC has an endothelial-protective effect in atherosclerosis. In addition, the blood ucOC and OC levels were negatively associated with chronic inflammation parameters, such as CRP, in patients with type 2 diabetes [116]. OC could be a therapeutic target for protecting against chronic inflammation and adverse events. Moreover, there is an interesting report concerning diabetic cardiomyopathy. UcOC may have a promising effect in improving microvascular insufficiency and myocardial dysfunction in diabetic cardiomyopathy [117]. Diabetic cardiomyopathy is accompanied by microvascular complications that lead to myocardial dysfunction and heart failure [118]. Most conventional therapies cannot ameliorate the microvascular insufficiency in diabetic cardiomyopathy [119]. Both the systolic and diastolic dysfunctions observed in rat models using streptozotocin were significantly improved after the ucOC levels were increased by the administration of warfarin [117]. Warfarin prevents posttranslational carboxylation of OC, resulting in increased blood levels of ucOC [80]. Therefore, the role of ucOC as a therapeutic agent in the modulation of microvascular insufficiency and the subsequent cardiac functional and histopathological changes occurring after the complete establishment of diabetic myocardiopathy.

It was indicated that the blood OC levels are decreased in postmenopausal women with diabetes [120]. Although the blood level of OC increased in postmenopausal women and patients with osteoporosis, diabetes could induce disrupted bone metabolism. However, several prospective cohort studies reported the association of baseline blood OC levels and risk of incident diabetes even in postmenopausal women [103,104]. Furthermore, a previous study reported that patients treated with bisphosphonates had low-incident risk of diabetes [121]. Because bisphosphonate treatment for osteoporosis reduces bone turnover, it is possible that serum OC levels are reduced. However, it was reported that there was no trend in the increase of incident diabetes rate in subjects with bisphosphonate treatment compared to subjects without treatment for osteoporosis [103]. Therefore, it could be considered that there are some unknown mechanisms independent with reduction in OC.

## 4. Sclerostin

### 4.1. The Regulation of Glucose Metabolism by Sclerostin

Sclerostin is mainly expressed in osteocytes and suppresses bone formation via the inhibition of the Wnt-low-density-lipoprotein receptor-related protein (LRP) 5/6 signaling pathway on osteoblasts [122]. Sclerostin has already become the target of a new drug for osteoporosis and currently anti-sclerostin monoclonal antibody is used to treat severe osteoporosis [123,124,125]. Furthermore, it was also proposed that sclerostin involves negative regulation of glucose metabolism through upregulating adiposity, because activators of the canonical Wnt pathway have been implicated as potent inhibitors for adipogenesis [126]. In humans, it was reported that the blood sclerostin level is positively associated with fat mass [127,128]. It was also reported that increased blood sclerostin level is significantly associated with insulin resistance in skeletal muscle, liver, and adipose tissue in patients with diabetes [129]. In an experimental study, systemic sclerostin knockout mice showed increased bone mass, decreased fat mass, and improved glucose metabolism [130]. The study also demonstrated that anti-sclerostin antibody treatment in wild-type mice decreased fat mass and enhanced adipocyte differentiation [130]. According to these results, in addition to osteoporosis, sclerostin could contribute to the treatment of obesity.

On the other hand, it was shown that circulatory sclerostin induces browning of white adipose tissue by inhibiting Wnt-LRP5/6 signaling in an experimental model [15]. Although both osteocytic and blood sclerostin levels were increased in mice lacking the *stimulatory subunit of G-proteins* (*Gsα*) in osteocytes, beige adipogenesis was increased in white adipose tissue with the suppression of Wnt-LRP5/6 signaling [15]. These mice showed decreased body weight gain and fat mass [15]. In addition, the administration of sclerostin increased the brown adipose tissue specific gene, *uncoupling-protein 1* (*Ucp-1*), in white adipose tissue of wild-type mice by inhibiting Wnt-LRP5/6 signaling [15]. Furthermore, anti-sclerostin antibody treatment in *Gsα* knockout mice decreased high expression of *Ucp-1* in white adipose tissue [15]. In addition to an experimental study, a cohort study did not find an association between the blood sclerostin level and the incidence of type 2 diabetes in a non-diabetic population [131]. Therefore, further investigation concerning the detailed function of sclerostin or Wnt-LRP5/6 signaling in adipogenesis is required.

### 4.2. Sclerostin in Diabetic Complications

The association of increases in blood sclerostin level with diabetic cardiovascular diseases has been reported. It was reported that the blood sclerostin level can become reliable diagnostic and prognostic biomarker of cardiovascular risk in patients with type 2 diabetes [132]. Another report indicated that the blood sclerostin level could serve as a useful biomarker of early atherosclerosis in obese individuals without a previous history of cardiometabolic disorders [133]. Furthermore, there is a positive association between the blood sclerostin level and carotid intima media thickness (CIMT) [134,135]. In addition, the blood level of sclerostin is positively associated with the carotid-femoral pulse wave velocity (cfPWV) and independently predicts aortic stiffness in patients with type 2 diabetes [136]. Canonical Wnt signaling pathway activation is implicated in pro-proliferation in arterial and venous vascular smooth muscle cells [137]. In addition, the Wnt pathway has been reported to play an important role in the regulation of endothelial inflammation, vascular calcification, and mesenchymal stem cell differentiation [138]. Therefore, it is suggested that sclerostin is not only involved in the regulation of bone formation but also in the pathophysiological process of atherosclerosis. In patients with type 2 diabetes, a deleterious effect on trabecular bone architecture and cortical bone geometry is observed due to the lower bone formation and higher bone resorption. It was reported that the alterations in microarchitecture and bone turnover associated with type 2 diabetes are not a consequence of the elevated expression of sclerostin in an animal model of diabetes [139].

## 5. Lipocalin 2

### 5.1. The Regulation of Glucose Metabolism by Lipocalin 2

Lipocalin 2 is also termed 24p3 or neutrophil gelatinase-associated lipocalin (NGAL) [140]. Lipocalin 2 was understood as one of the adipokines [141]; however, a recent report indicated that lipocalin 2 is more highly expressed on osteoblasts in comparison to adipocytes [16]. They also found that mice with the conditional knockout of lipocalin 2 in osteoblasts showed increased blood glucose levels and body fat in mice [16]. Furthermore, it was revealed that lipocalin 2 secreted by osteoblasts binds to melanocortin receptor 4 (MC4R) in the paraventricular and ventromedial neurons of the hypothalamus and directly suppresses appetite [16]. Supporting this model, feeding increased the osteoblastic and blood lipocalin 2 and suppressed the food intake of wild-type mice [16]. In addition, the continuous administration of lipocalin 2 to wild-type mice decreased their food intake, fat mass, body weight and improved their glucose metabolism and energy expenditure [16]. Adding to experimental investigations, they described that the blood lipocalin 2 level was inversely associated with body weight and HbA1c in patients with type 2 diabetes [16]. On the other hand, one report suggested that the global ablation of lipocalin 2 did not affect glucose metabolism and body weight gain in mice [142]. In clinical studies, higher blood lipocalin 2 levels are associated with obesity, insulin resistance, and dyslipidemia in patients with type 2 diabetes [143,144]. In pathological states, the expression of lipocalin 2 was induced in broad organs such as kidney and liver [145,146]. Therefore, blood lipocalin 2 level is a biomarker for metabolic disorders. To resolve these discrepancies, metabolic functions of bone-derived lipocalin 2 and lipocalin 2 secreted from other tissues are further identified. Although further investigation is needed in both experimental and clinical studies, lipocalin 2 is expected to be a new therapeutic target to control appetite and obesity.

### 5.2. Lipocalin 2 in Diabetic Complications

To date, the relationship between bone-derived lipocalin 2 and diabetic complications have not been elucidated. In clinical studies, it was demonstrated that increased systemic levels are associated with severity of coronary artery disease and increased risk of incidence cardiovascular diseases [147,148]. Further studies are required.

## 6. Future Directions

As an endocrine organ, bone has various effects on glucose metabolism and diabetic complications through bone-derived hormones. However, several issues remain to be clarified. There may be differences in the bone and glucose metabolism of mice and humans. Furthermore, in vitro experiments cannot mimic in vivo environments. In particular, the pathogenesis of diabetes is much more complicated in humans, involving multiple genetic and environmental factors, including social, educational, and economic status [149]. Therefore, more solid human evidence from longitudinal and prospective studies are required. Cross-sectional studies cannot induce cause-and-effect relationships. We believe that the potential applications of bone-derived hormones in the treatment of diabetes are worthy of further study and hope that other new antidiabetic drugs emerge. Furthermore, there are possibly new bone-derived hormones to be identified. If proven to also be true in humans, bone will be established as a new player in diabetes and diabetic complications.

## 7. Conclusions

The functions of bone extend beyond supporting the body, protecting the internal organs and the central nervous system, and its contribution to hematopoiesis. Bone acts as an endocrine organ to secrete several hormones and regulate functions of other organs through the formation of a multi-organ network system. Especially, the involvement of several bone-derived hormones in glucose metabolism and diabetic complications has been identified. We believe that further investigations concerning the network system among multiple organs via bone-derived hormones may give us a new perspective that facilitates the prediction, prevention, and treatment of diabetes and its complications.

## Figures and Tables

**Table 1:** Summary of bone-derived hormones.

Hormone	Target Organ	Receptor	Function
FGF23	kidney	FGFR1c/α-Klotho complex	reduce blood phosphate level
ucOC	pancreas	GPRC6A	increase insulin secretion
	adipose tissue		increase adiponectin secretion, reduce fat mass
	testis		increase testosterone secretion
	intestine		increase GLP-1 secretion
	muscle		increase exercise capacity
	brain	GPR158	increase cognitive function
Sclerostin	bone	Wnt-LRP5/6	reduce bone formation
	adipose tissue		induce browning of white adipose tissue
	peripheral tissue	Unknown	increase insulin resistance
Lipocalin 2	brain	MC4R	reduce appetite

FGF, fibroblast growth factor; ucOC, undercarboxylated osteocalcin; FGFR, FGF receptor; GPRC6A, G protein-coupled receptor, class C, group 6, subtype A; GPR158, G protein-coupled receptor 158; LRP5/6, low-density lipoprotein receptor-related protein 5/6; MC4R, melanocortin 4 receptor; GLP-1, glucagon-like peptide-1.

## Data Availability

Not applicable.
